# Magnesium Silicate-Based Cement with Moderated Alkalinity: Physicochemical Characterization and Human Dental Pulp Cell Responses

**DOI:** 10.3390/jfb17100502

**Published:** 2026-10-04

**Authors:** Yoon Seon Lee, HyeRyoung Wang, Yu-Jin Ahn, So Young Park, Jihoon Lee, Jin Man Kim, Juhyuk Moon, Won Jun Shon

**Affiliations:** 1 Department of Conservative Dentistry, School of Dentistry and Dental Research Institute, Seoul National University, Seoul 03080, Republic of Korea; seon1663@snu.ac.kr (Y.S.L.); hrwang@snu.ac.kr (H.W.); yuahn@snu.ac.kr (Y.-J.A.); 2 Department of Oral Microbiology and Immunology, School of Dentistry and Dental Research Institute, Seoul National University, Seoul 08826, Republic of Korea; sypark1004@snu.ac.kr (S.Y.P.); jinmankim@snu.ac.kr (J.M.K.); 3 Department of Civil and Environmental Engineering and Institute of Construction and Environmental Engineering, Seoul National University, Seoul 08826, Republic of Korea; basic730@snu.ac.kr

**Keywords:** magnesium silicate-based cement, endodontic biomaterial, alkalinity, human dental pulp cells, calcium signaling

## Abstract

To establish a lower-alkalinity alternative to calcium silicate-based endodontic materials, this study developed a self-setting magnesium silicate-based cement (MSC) that hardens through magnesium silicate hydrate (M-S-H) formation. MSC was formulated from magnesium oxide, reactive silica, and zirconium oxide. Hydration and phase evolution were characterized by X-ray diffraction, thermogravimetric analysis, thermodynamic modeling, and scanning electron microscopy. pH, early penetration resistance, and compressive strength were compared with ProRoot mineral trioxide aggregate (MTA), and cement extracts were evaluated for human dental pulp cell viability, alkaline phosphatase activity, intracellular calcium mobilization, and YAP localization. MSC hydration involved progressive Mg(OH)_2_ consumption and formation of an M-S-H-rich matrix. Its pH decreased from approximately 9.7 to 8.9, whereas MTA remained near pH 12. The lower-water formulation containing additives exceeded 40 MPa in compressive strength at 28 days and showed greater early penetration resistance. MSC-0.3 extracts showed higher 7-day cell viability than ProRoot MTA at 1:4 and 1:8 dilutions, increased alkaline phosphatase activity, were accompanied by increased intracellular calcium fluorescence that was not observed following NPS 2143 pretreatment, and increased nuclear YAP localization. These findings identify MSC as a functional cement platform combining moderated alkalinity, progressive strength development, and favorable early pulp-cell responses.

## 1. Introduction

Bioactive endodontic materials can support dentin–pulp repair by releasing biologically active ions and modulating mineralization-associated responses in dental pulp cells [1,2]. Among these materials, hydraulic calcium silicate-based cements are widely used in vital pulp therapy because they hydrate in the presence of water and promote hard-tissue barrier formation following pulp exposure [3,4,5]. Mineral trioxide aggregate (MTA), a representative hydraulic calcium silicate-based cement, is currently considered the clinical gold standard due to its excellent sealing ability and proven hard-tissue induction [6,7,8,9]. However, the hydration of MTA—primarily involving tricalcium silicate (C_3_S) and dicalcium silicate (C_2_S)—inevitably produces calcium hydroxide (Ca(OH)_2_), resulting in a highly alkaline environment (pH > 12) [3,10]. While this alkalinity provides antimicrobial benefits, it can also induce caustic stress on surrounding dental pulp cells, potentially leading to tissue necrosis or impaired regenerative performance, particularly in cases with compromised wound healing potential [11,12,13].

Magnesium (Mg^2+^) is an essential cofactor for over 300 enzymatic reactions, including those vital for DNA and protein synthesis [14]. Within mineralized dental tissues, it is naturally incorporated into the hydroxyapatite structure, comprising approximately 0.25–0.5% of enamel and up to 1.2% (*w*/*w*) of dentin, where it plays a key role in modulating crystal growth and lattice stability during early biomineralization [15,16]. These properties have prompted interest in magnesium-containing biomaterials, particularly because magnesium-rich microenvironments may enhance odontogenic responses in human dental pulp cells [17,18]. However, previous approaches have largely relied on soluble magnesium ions, doped bioactive glasses, or scaffold systems rather than self-setting cements suitable for endodontic repair [19,20,21]. A self-setting, solid-phase magnesium silicate-based cement may therefore provide a mechanically stable platform for exploring magnesium-associated bioactivity in endodontic applications. Unlike magnesium-based approaches relying on soluble ion supplementation, doped bioactive glasses, or preformed scaffolds, a MgO–reactive silica cement can generate an M-S-H-rich binding phase directly through aqueous hydration. This reaction pathway provides a means of achieving self-setting behavior without the substantial Ca(OH)_2_ production characteristic of calcium silicate cements, thereby allowing structural hardening and alkalinity to be modulated through a distinct binder chemistry.

In this study, we developed an experimental magnesium silicate-based cement (MSC) as an alternative to conventional calcium silicate-based systems. The material was designed to form a magnesium silicate hydrate (M-S-H)-rich matrix that moderates the excessive alkalinity associated with conventional hydraulic cements while supporting cement hardening and mechanical development. Its physicochemical characteristics were evaluated, and the selected MSC-0.3 formulation was assessed for human dental pulp cell viability and alkaline phosphatase activity. Intracellular Ca^2+^ mobilization and YAP localization were further examined as exploratory signaling outcomes, given the reported crosstalk between Ca^2+^ and Hippo–YAP signaling and the involvement of YAP in odontogenic differentiation [22,23]. We hypothesized that magnesium silicate hydration would provide a balanced cement platform combining moderated alkalinity with favorable physicochemical performance and early biological responses.

## 2. Materials and Methods

### 2.1. Cement Preparation

An experimental magnesium silicate-based cement (MSC) was prepared from EP-grade magnesium oxide (MgOG), reactive silica (RS), and zirconium oxide (ZrO_2_); the chemical composition of RS is listed in Table 1. ProRoot MTA (Dentsply Sirona Inc, Charlotte, NC, USA) served as the calcium silicate-based cement control and was mixed for 2 min at a water-to-binder ratio of 0.34, according to the manufacturer’s instructions. For MSC preparation, dry powders containing 14 g MgO, 56 g RS, and 30 g ZrO_2_ were mixed for 1 min. Two formulations were prepared; MSC-0.5 was mixed with 50 g deionized water, whereas MSC-0.3 was mixed with 30 g deionized water containing 5.5 g polycarboxylate ether-based superplasticizer (SP; Flowmix 3000U, Dongnam, Gyeonggi-do, Republic of Korea) and 0.5 g triethanolamine (TEA; Daejung Co., Ltd., Gyeonggi-do, Republic of Korea). A polycarboxylate ether-based SP was selected because PCE dispersants adsorb on silica surfaces and promote particle dispersion through electrostatic repulsion and steric hindrance, thereby improving fluidity in low-water, silica-containing cementitious systems [24]. TEA was incorporated to mitigate the delayed early stiffening associated with the SP-containing formulation [25,26]. The additives were dissolved in deionized water before mixing with the powder blend. MSC-0.5 and MSC-0.3 were designated according to nominal added-water-to-total-powder ratios of 0.50 and 0.30, respectively, calculated using the combined mass of MgO, RS, and ZrO_2_ as the dry-powder denominator. SP and TEA were excluded from this nominal ratio. Pastes were mixed for 2 min, molded (5 mm per side; stainless steel, Teflon, or acrylic depending on the test type), and cured at 37 °C and 99% relative humidity. After 1 day, the samples were demolded and further cured under the same environmental conditions. Mixture proportions are listed in Table 2.

### 2.2. Physicochemical Characterization

#### 2.2.1. X-Ray Diffraction (XRD)

Hydration products and phase evolution were evaluated after 1, 3, 7, and 28 days of curing. Specimens were ground into powder after mechanical testing, and hydration was stopped by solvent exchange using isopropyl alcohol followed by ethyl ether. X-ray diffraction was performed using a D2 PHASER (Bruker AXS GmbH, Karlsruhe, Germany) with Cu-Kα radiation (λ = 1.5418 Å) over a 2θ range of 6–60°. Phase identification and quantitative analysis were performed by Rietveld refinement using X’pert HighScore Plus software (v4.8, PANalytical, Amelo, The Netherlands), referencing the Inorganic Crystal Structure Database (ICSD) [27] and the Crystallography Open Database (COD) [28]. TiO_2_ was used as an internal standard to estimate crystalline and amorphous phase contents [29]. Sample mass was normalized to a dry-mass basis using the mass remaining at 550 °C ($w_{550{}^{\circ}C}$), as described previously [30].

$$w_{dry}=\frac{w_{measured}}{1-H_{2}O_{bound}}=\frac{w_{measured}}{w_{550{}^{\circ}C}}$$

#### 2.2.2. Thermogravimetric Analysis

Thermogravimetric analysis was performed using an SDT Q600 (TA Instruments, New Castle, DE, USA) with approximately 25 mg of sample heated from 20 °C to 1000 °C at 10 °C/min. Differential thermogravimetric curves were used to identify weight-loss events associated with magnesium silicate hydrate dehydration and magnesium hydroxide dehydroxylation. Chemically bound water was defined as the cumulative mass loss up to 550 °C.

#### 2.2.3. Thermodynamic Modeling

The phase assemblage and pore solution pH were calculated using a Gibbs free-energy minimization program, GEM-Selektor v.3.9.5 [31,32]. For the modeling, the Cemdata 18 [33] and the built-in thermodynamic database were used [34]. The extended Debye–Hückel equation was applied to calculate the activity coefficients for aqueous species in the cement system, as follows [35]:

$$\log_{10}\gamma_{i}=\;\frac{-A_{\gamma }z_{i}^{2}\sqrt{I}}{1+\dot{a}B_{\gamma }\sqrt{I}}\;+\;b_{\gamma }I\;+\log_{10}\frac{X_{jw}}{X_{w}}$$

where γ_i_ and z_i_ denote the activity coefficient and charge of the ith aqueous species, respectively; A_γ_ is a temperature coefficient; B_γ_ is a pressure coefficient; I denotes the molar ionic strength of the pore solution; X_jw_ is the molar quantity of water; X_w_ is the total molar amount of the aqueous phase; $\dot{a}\;$and B_γ_ are the common ion-size parameter and short-range interaction parameter, respectively. $\dot{a}\;$was set to 3.67 Å and B_γ_ was determined to be 0.123 kg/mol. The temperature and pressure were set to 37 °C and 1 bar, respectively.

#### 2.2.4. pH Measurement

For pH measurements, disks (10 mm × 2 mm) were immersed individually in 10 mL deionized water at 37 °C. Fresh specimens and solutions were used at 0, 3, 7, 14, and 21 days, and pH was measured using a calibrated Orion Star A211 Benchtop pH Meter (Thermo Fisher Scientific, Waltham, MA, USA). To prevent potential buffering effects and ensure independent measurements, separate specimens and fresh solutions were prepared for each designated time point.

#### 2.2.5. Compressive Strength and Early Penetration Resistance

Compressive strength was measured after 1, 3, 7, and 28 days using 5-mm cubic specimens and an Instron 3345 universal testing machine (Instron, Norwood, MA, USA) at 0.1 mm/min. Specimens were cured at 37 °C and 99% relative humidity, demolded after 24 h, and surface-dried before testing. Early penetration resistance was assessed 5 min after the start of mixing using an automatic Vicat apparatus (HJ-1116M, MATEST, Treviolo, Italy) in an EN 196-3-based screening procedure [36]. A penetration depth of <1 mm was predefined as a study-specific comparative threshold and was not regarded as a standardized or clinically validated setting-time criterion for endodontic materials.

#### 2.2.6. Scanning Electron Microscopy

Scanning electron microscopy (SEM) was performed to evaluate the microstructure of the samples. Specimens were dried and sputter-coated with gold prior to SEM observation. Finally, the specimens were examined using a scanning electron microscope (S-4700, Hitachi, Tokyo, Japan). Representative elemental analyses of MSC-0.3 and ProRoot MTA specimens after 3 and 14 days of hydration were performed by EDS coupled to the SEM, using the matrix regions indicated in Figure S1.

### 2.3. Cell Culture and Cement Extract Preparation

Following physicochemical formulation screening, MSC-0.3 was selected as the lead formulation for subsequent biological assays. Accordingly, all cell-based experiments were performed using MSC-0.3 and ProRoot MTA extracts. Primary hDPCs were obtained from three donors aged 18–22 years who provided written informed consent; the cells were used at passages 1–3. The study protocol was approved by the Institutional Review Board of Seoul National University (IRB No: S-D20140007). Each donor constituted one independent experiment, with three technical replicates per condition. Cells were cultured in α-MEM containing 10% FBS, 100 U/mL penicillin, and 100 µg/mL streptomycin at 37 °C and 5% CO_2_ and used at 70–80% confluence. Set MSC-0.3 and ProRoot MTA specimens were extracted in culture medium at 37 °C for 24 h according to ISO 10993-12 [37]. The original extract was designated 1:1 and diluted with fresh culture medium to obtain 1:2, 1:4, and 1:8 extracts.

### 2.4. Cell Viability and Alkaline Phosphatase Activity

For the MTT assay, hDPCs were seeded at 1 × 10^4^ cells/well, attached for 24 h, and exposed to cement extracts or control medium. After 1, 3, or 7 days, MTT was applied for 4 h, formazan was dissolved in dimethyl sulfoxide, and absorbance was measured at 570 nm. Values were expressed relative to untreated controls.

For ALP analysis, cells were seeded at 1 × 10^5^ cells/well in odontogenic differentiation medium containing cement extracts. ALP activity was measured on days 3, 7, and 14 to capture representative early, intermediate, and later differentiation-associated responses, using p-nitrophenyl phosphate, normalized to total protein, and confirmed by staining on day 7.

### 2.5. Intracellular Calcium Mobilization and YAP Localization

Human dental pulp cells were transfected with G-GECO1, a genetically encoded calcium indicator. After transfection and serum starvation, cells were exposed to cement extracts, and real-time fluorescence changes were recorded by confocal microscopy (LSM 700, Carl Zeiss, Jena, Germany). To assess the involvement of the calcium-sensing receptor, cells were pretreated with NPS 2143 before exposure to the MSC-0.3 extract. Real-time G-GECO1 fluorescence was quantified from individually tracked cells using Zen software v3.4 and normalized to the pretreatment baseline. The resulting time-course traces were summarized descriptively.

After 7 days, nuclear and cytoplasmic YAP levels were quantified by Western blotting and normalized to Lamin B and GAPDH, respectively.

### 2.6. Statistical Analysis

For compressive-strength, pH, and 5-min Vicat penetration measurements, technical replicates were averaged within each of three independently prepared cement batches, and each batch mean was treated as one independent observation (*n* = 3). For cell viability, ALP activity, and YAP analyses, technical replicates were averaged within each donor, and each donor was treated as the independent experimental unit (*n* = 3). Quantitative data are presented as mean ± standard deviation unless otherwise specified. Statistical analyses were performed using IBM SPSS Statistics, version 25.0 (IBM Corp., Armonk, NY, USA).

Compressive strength and pH were analyzed using two-way ANOVA. Cell viability was analyzed separately at each time point using two-way repeated-measures ANOVA, and ALP activity was analyzed using two-way repeated-measures ANOVA. Nuclear and cytoplasmic YAP levels were analyzed separately using one-way repeated-measures ANOVA. Bonferroni-adjusted pairwise comparisons were performed where appropriate. The 5-min Vicat penetration measurements and calcium-fluorescence time courses were summarized descriptively.

Effect sizes for ANOVA models are reported as partial eta squared (partial η^2^). Complete descriptive statistics and primary statistical outcomes are provided in Supplementary Tables S1 and S2. All statistical tests were two-sided, and *p* < 0.05 was considered statistically significant.

## 3. Results

### 3.1. Phase Evolution and Thermal Characteristics

**Figure 1:**
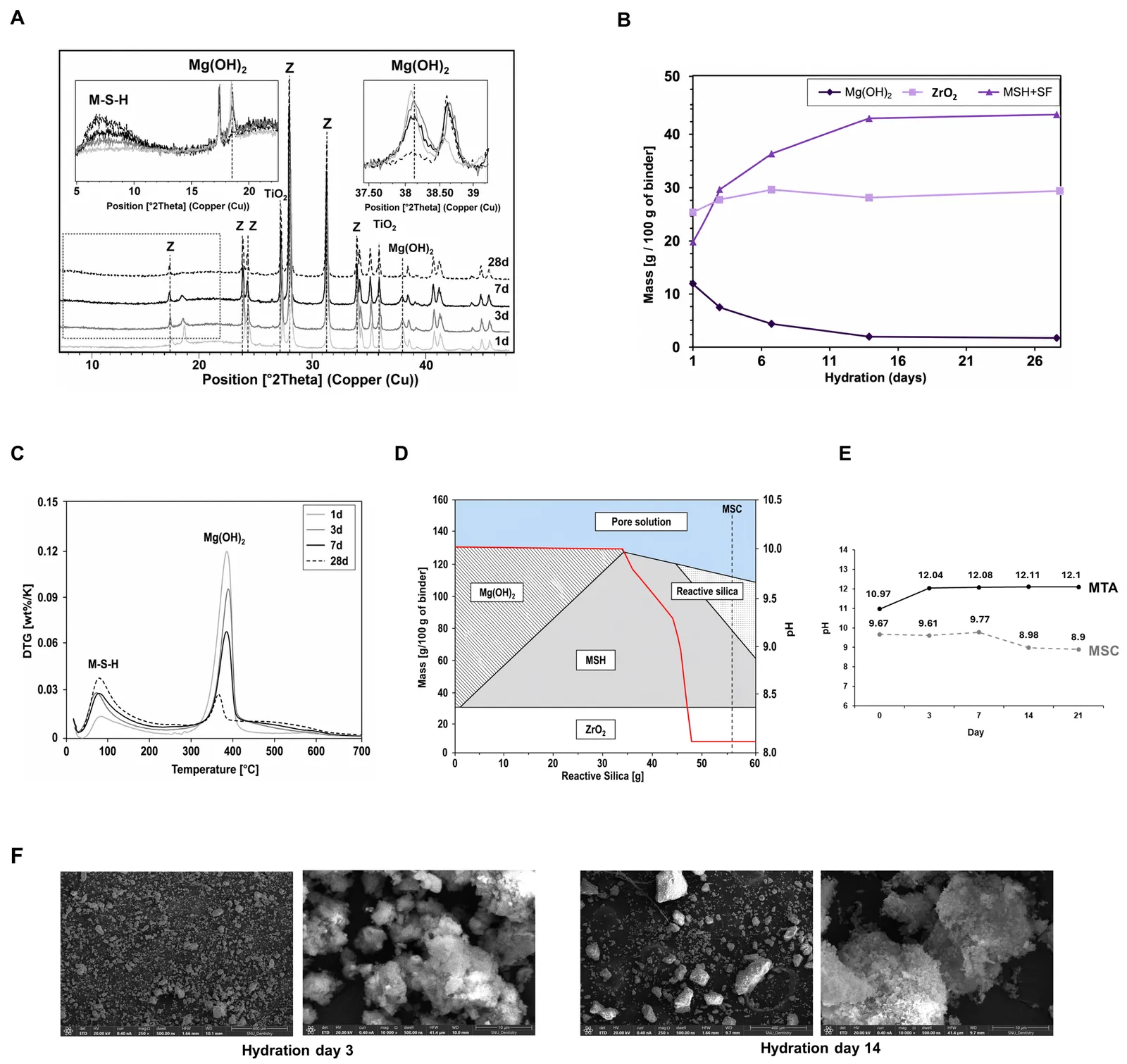
Physicochemical characterization and hydration behavior of MSC paste. (**A**) X-ray diffraction patterns after 1, 3, 7, and 28 days of hydration; insets show the amorphous region and Mg(OH)_2_ reflections. (**B**) Quantitative phase analysis by Rietveld refinement at each hydration time point. (**C**) Differential thermogravimetric curves obtained after 1, 3, 7, and 28 days of hydration. (**D**) Thermodynamic modeling of phase assemblage and pore-solution pH as a function of reactive silica content; the red line represents the modeled pore-solution pH. (**E**) Experimental pH values of MSC and ProRoot MTA over time. (**F**) Scanning electron microscopy images of MSC after 3 and 14 days of hydration. Scale bars: 400 and 10 μm.

Overall, MSC showed progressive hydration over time, characterized by the consumption of Mg(OH)_2_, formation of an amorphous magnesium silicate hydrate (M-S-H)-rich phase, and gradual development of a more integrated microstructure. After 1 day of hydration, distinct peaks for brucite (Mg(OH)_2_) were detected at approximately 18.5° and 38° 2θ. Notably, the intensity of these brucite peaks gradually decreased over time, particularly from day 7 to day 28. Concurrently, a broad amorphous hump appeared at low 2θ angles (5–15°), which became more pronounced as hydration progressed (Figure 1A). Quantitative phase analysis by Rietveld refinement confirmed a time-dependent reduction in Mg(OH)_2_ and a corresponding increase in the amorphous fraction, whereas the ZrO_2_ content remained relatively stable throughout the experimental period (Figure 1B).

To further validate the hydration kinetics through a complementary analytical method, differential thermogravimetry (DTG) was performed (Figure 1C). A low-temperature weight-loss peak at approximately 100 °C was attributed to dehydration of the M-S-H-rich phase, whereas the broader peak between 350 and 420 °C was associated with dehydroxylation of Mg(OH)_2_. Over time, the M-S-H-associated peak increased, whereas the Mg(OH)_2_-associated peak decreased.

### 3.2. Thermodynamic Modeling, pH Behavior and Microstructural Development

Thermodynamic modeling predicted progressive M-S-H formation and Mg(OH)_2_ depletion with increasing reactive silica content, accompanied by a decrease in calculated pore-solution pH (Figure 1D). No detectable phase transformation or consumption of ZrO_2_ was observed during hydration, while M-S-H was formed at the expense of Mg(OH)_2_ consumption. According to the simulation, a sharp decline in the pore solution pH occurs once Mg(OH)_2_ is fully depleted, declining to a mildly alkaline range of approximately pH 8.1.

The experimental pH showed the same overall downward trend as the thermodynamic prediction but did not reach the modeled equilibrium value. The pH of MSC-0.3 showed an overall decrease from 9.67 ± 0.16 at the initial measurement to 8.90 ± 0.15 at day 21. In contrast, ProRoot MTA increased from 10.97 ± 0.16 initially to 12.04 ± 0.21 at day 3 and remained near pH 12 thereafter (Figure 1E). The higher experimental pH likely reflects the persistence of residual Mg(OH)_2_ and the different boundary conditions of the equilibrium pore-solution model and the external immersion measurement. The pH profiles differed significantly between materials over time; complete statistical outcomes are provided in Supplementary Tables S1 and S2.

Furthermore, scanning electron microscopy revealed time-dependent changes in the microstructure of MSC (Figure 1F). At 3 days, the hydrated paste displayed a relatively porous and loosely connected structure. By 14 days, the matrix appeared denser and more integrated, with greater continuity of the hydration products and reduced apparent porosity.

Representative EDS spectra obtained from MSC-0.3 matrix regions at 3 and 14 days detected Mg, Si, O, and Zr (Figure S1). The corresponding local Mg/Si atomic ratios were approximately 0.86 and 0.63, respectively, supporting the presence of a Mg–Si-rich reaction matrix. In contrast, ProRoot MTA showed a Ca–Si–O-rich elemental profile with detectable Bi. Because EDS provides local elemental composition rather than structural phase identification, these findings were interpreted as complementary evidence supporting, rather than independently confirming, formation of an M-S-H-rich amorphous phase.

### 3.3. Mechanical Properties and Early Penetration Resistance

**Figure 2:**
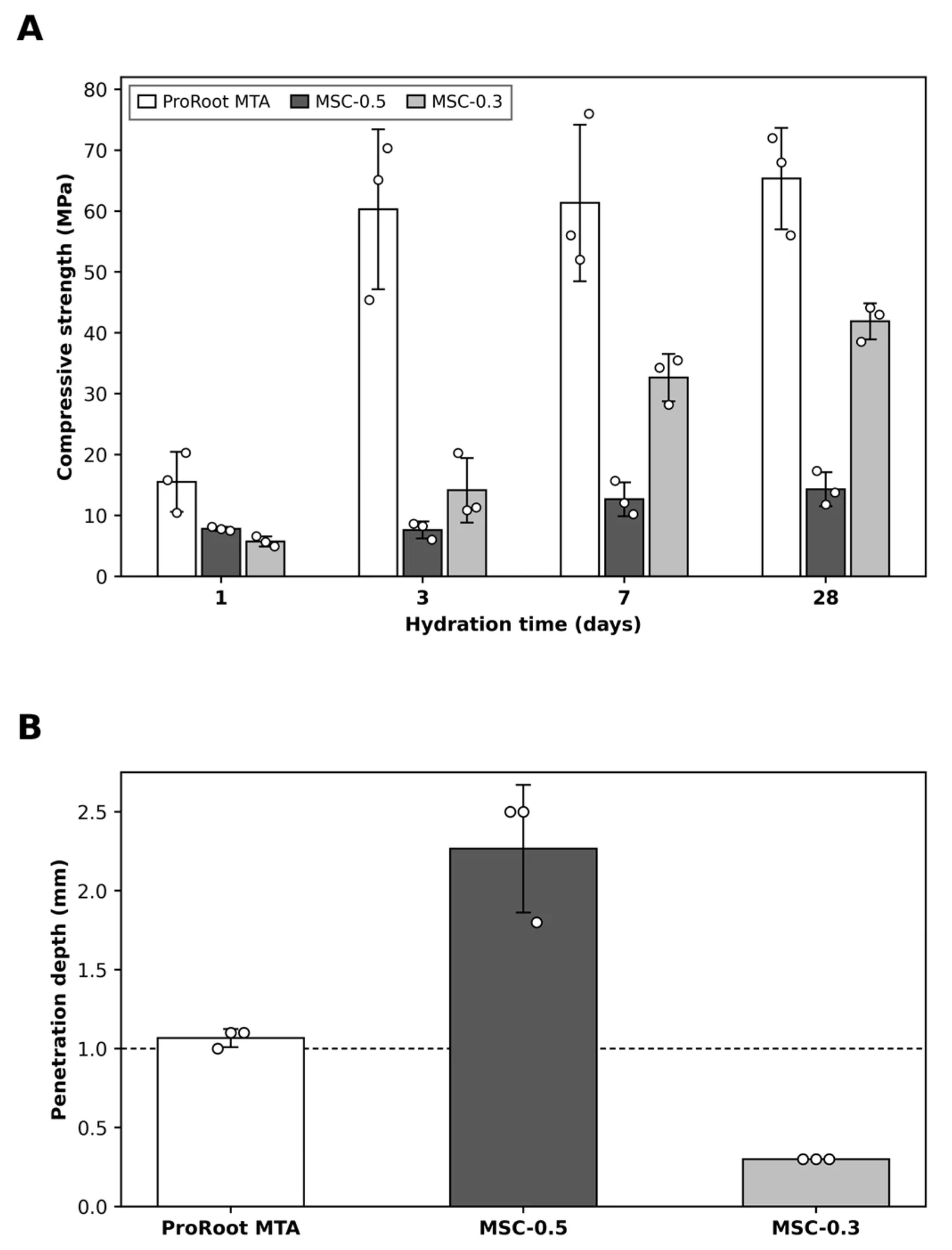
(**A**) Compressive strength of ProRoot MTA, MSC-0.5, and MSC-0.3 after 1, 3, 7, and 28 days of hydration. (**B**) Early penetration depth measured 5 min after the start of mixing. Data are presented as mean ± standard deviation from three independently prepared cement batches (*n* = 3), with technical specimens averaged within each batch; individual points represent batch means. The dashed line in panel B denotes the study-specific 1-mm threshold used for comparative formulation screening and does not represent a standardized or clinically validated setting-time criterion.

The mechanical performance of MSC was strongly formulation-dependent (Figure 2A). ProRoot MTA showed a marked early increase in compressive strength, from 15.5 ± 4.9 MPa at day 1 to 60.3 ± 13.2 MPa at day 3, and remained relatively stable thereafter, reaching 65.3 ± 8.3 MPa at day 28. MSC-0.3, prepared at a nominal added-water-to-total-powder ratio of 0.30 with SP and TEA, showed progressive strength development from 5.7 ± 0.8 MPa at day 1 to 41.9 ± 3.0 MPa at day 28. In contrast, MSC-0.5, prepared at a nominal added-water-to-total-powder ratio of 0.50 without these additives, increased only from 7.8 ± 0.3 MPa to 14.3 ± 2.8 MPa over the same period. The slower but continuous strength development of MSC was consistent with the lower reactivity of RS relative to alite (C_3_S) and the prolonged formation of the M-S-H matrix [38,39,40]. The continued strength gain of MSC-0.3 also paralleled the progressive consumption of Mg(OH)_2_ and increase in the M-S-H-rich amorphous fraction observed during hydration. Because water content and additive composition varied simultaneously, the greater strength development of MSC-0.3 should be interpreted as a formulation-level effect rather than as the independent effect of any single component. Compressive-strength development differed significantly among materials over time; complete statistical outcomes are provided in Supplementary Tables S1 and S2.

Vicat penetration depth also differed markedly among formulations (Figure 2B). At 5 min after mixing, MSC-0.3 exhibited the lowest penetration depth, falling below the predefined 1-mm threshold, whereas MSC-0.5 remained above 2 mm and ProRoot MTA was close to 1 mm. Together, these findings show that the lower-water formulation containing SP and TEA exhibited greater early penetration resistance and subsequent compressive strength than the higher-water formulation without these additives.

### 3.4. Biological Responses and Intracellular Signaling of hDPCs

#### 3.4.1. Cytocompatibility and ALP Activity

**Figure 3:**
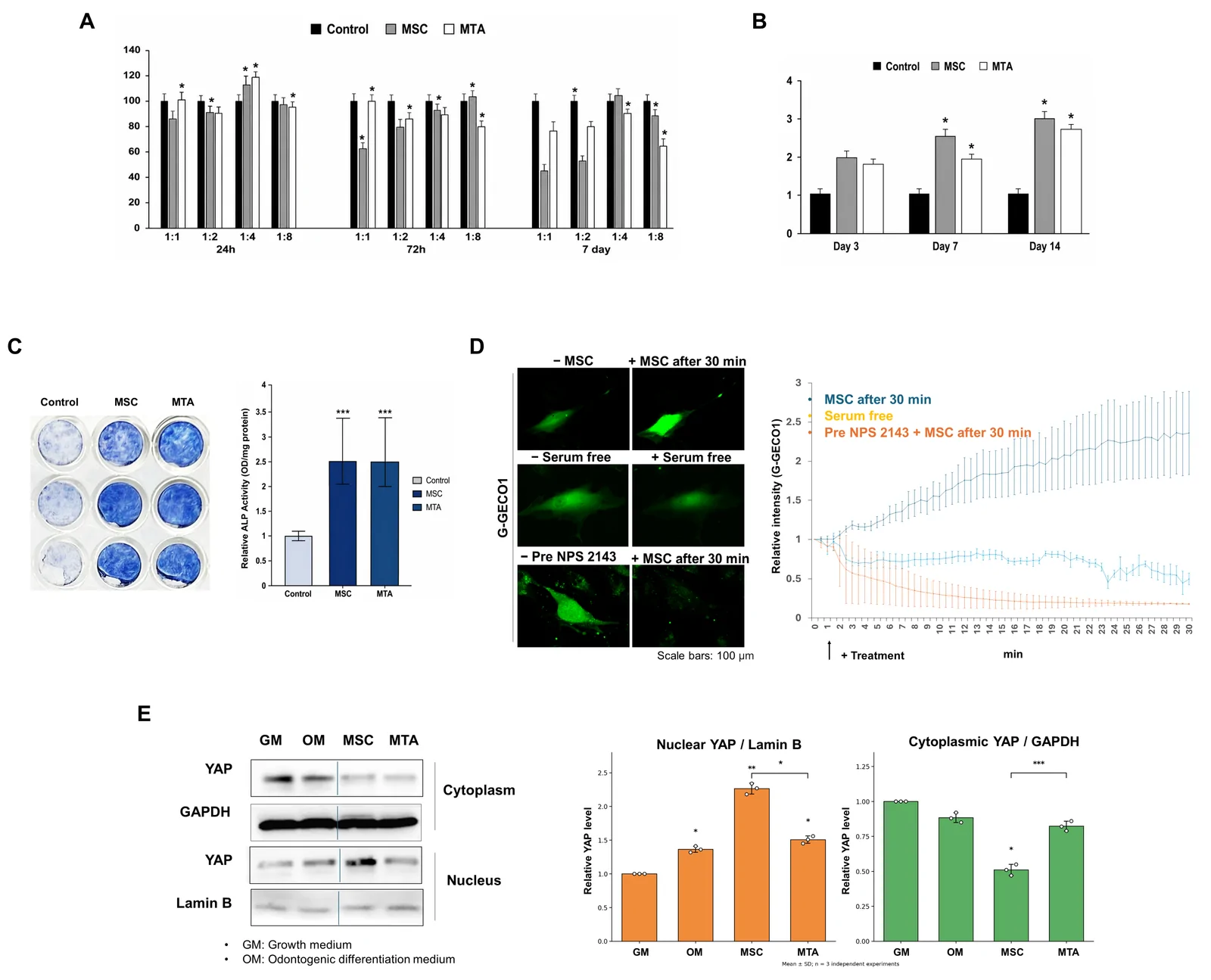
Cytocompatibility, ALP activity, intracellular Ca^2+^ signaling, and YAP localization in human dental pulp cells treated with MSC-0.3 extracts. (**A**) Cell viability assessed by MTT assay after 24 h, 72 h, and 7 days using extract dilutions of 1:1, 1:2, 1:4, and 1:8. (**B**) ALP activity measured on days 3, 7, and 14. (**C**) Representative ALP staining and corresponding quantitative analysis on day 7. (**D**) Representative G-GECO1 fluorescence images and descriptive time-course analysis under MSC-0.3 treatment (*n* = 4 tracked cells), serum-free conditions (*n* = 3 tracked cells), and NPS 2143 pretreatment followed by MSC-0.3 exposure (*n* = 3 tracked cells). Scale bars: 100 μm. The arrow indicates the time of treatment addition. (**E**) Western blot analysis and quantification of YAP in cytoplasmic and nuclear fractions under growth medium (GM), odontogenic differentiation medium (OM), MSC-0.3, and ProRoot MTA conditions. The intervening lane, representing a preliminary formulation-screening condition not included in the final biological comparison, was omitted. Vertical lines indicate noncontiguous lanes resulting from removal of the lane. For panels (**A**,**B**,**E**), data are presented as mean ± standard deviation from three donors (*n* = 3), with technical replicates averaged within each donor. Asterisks above the bars indicate comparisons with the control, whereas brackets indicate comparisons between the connected groups. * *p* < 0.05, ** *p* < 0.01, and *** *p* < 0.001.

Based on its lower early penetration depth and substantially greater compressive-strength development, MSC-0.3 was selected for the subsequent cell-based experiments. MSC-0.3 and ProRoot MTA showed time- and dilution-dependent effects on hDPC viability (Figure 3A). At 24 h, viability was generally maintained across the tested conditions, although differences were observed among individual dilutions. At 72 h, MSC-0.3 viability increased with extract dilution, ranging from approximately 63% at 1:1 to 103% at 1:8, whereas the response to ProRoot MTA was more variable. At 7 days, a crossover pattern was observed. ProRoot MTA maintained higher viability than MSC-0.3 at the more concentrated 1:1 and 1:2 conditions, whereas MSC-0.3 showed higher viability than ProRoot MTA at the more diluted 1:4 and 1:8 conditions. Cell-viability responses varied significantly according to material and extract dilution; complete statistical outcomes are provided in Supplementary Tables S1 and S2.

ALP activity increased over time in hDPCs exposed to MSC-0.3 extract (Figure 3B). It was higher than that of the control at days 7 and 14 and was comparable to or greater than that observed with ProRoot MTA. Consistent with the quantitative findings, alkaline phosphatase staining at day 7 was more intense in the experimental cement and ProRoot MTA groups than in the control group (Figure 3C). Quantitative analysis confirmed an approximately 2.5-fold increase in ALP activity in both the MSC-0.3 and ProRoot MTA groups relative to the control. Complete statistical outcomes are provided in Supplementary Tables S1 and S2.

#### 3.4.2. Intracellular Calcium Signaling and YAP Localization

To characterize intracellular responses to MSC-0.3 extract, Ca^2+^ mobilization and YAP localization were evaluated. MSC-0.3 exposure was accompanied by a sustained increase in relative G-GECO1 fluorescence over the 30-min observation period in the tracked cells, whereas no comparable increase was observed under serum-free conditions or following NPS 2143 pretreatment (Figure 3D). Western blot analysis further showed altered YAP distribution following MSC-0.3 treatment (Figure 3E). Nuclear YAP levels were increased, whereas cytoplasmic YAP levels were reduced compared with the control and ProRoot MTA groups. These findings were consistent with increased nuclear localization of YAP following MSC-0.3 treatment. Experimental conditions significantly affected both nuclear and cytoplasmic YAP levels; complete statistical outcomes are provided in Supplementary Tables S1 and S2.

## 4. Discussion

The development of bioactive materials capable of actively modulating the pulpal microenvironment is a central goal in regenerative endodontics. Although mineral trioxide aggregate (MTA) has become a clinical standard, its high alkalinity and potential for tooth discoloration have necessitated the search for alternatives. In the present study, the experimental magnesium silicate-based cement (MSC) maintained a moderately alkaline pH range and reached approximately 8.9, formed a mechanically stable magnesium silicate hydrate (M-S-H) matrix, and supported favorable early biological responses in hDPCs.

The hydration of MSC was governed by the reaction between magnesium oxide and reactive silica, resulting in progressive M-S-H formation. This pathway differs from that of conventional calcium silicate-based cements, which generate Ca(OH)_2_ and maintain a highly alkaline environment. Progressive consumption of Mg(OH)_2_ limited the accumulation of free hydroxide ions and was accompanied by pH stabilization. X-ray diffraction, Rietveld refinement, thermal analysis, and scanning electron microscopy collectively supported progressive formation of an M-S-H-rich matrix and gradual development of a more integrated microstructure. Representative local EDS measurements further supported the presence of a Mg–Si-rich reaction matrix, although EDS does not establish bulk Mg/Si stoichiometry or the definitive structural identity of the amorphous phase.

The relative viability of hDPCs depended on extract dilution rather than uniformly favoring either material. At 7 days, ProRoot MTA showed higher viability than MSC-0.3 at the more concentrated 1:1 and 1:2 dilutions, whereas MSC-0.3 showed higher viability at the more diluted 1:4 and 1:8 conditions. MSC-0.3 also enhanced alkaline phosphatase activity, consistent with an early mineralization-related or odontogenic response. This observation is biologically plausible given the roles of Mg^2+^ in mineralization and intracellular signaling [41,42,43] and prior evidence that magnesium-enriched environments promote odontogenic differentiation of human dental pulp cells [17]. Because biological testing was limited to MSC-0.3 and three young adult donors, formulation-dependent cellular responses and broader inter-donor variability require confirmation in subsequent studies. In addition, antimicrobial performance under the moderated alkaline conditions remains to be established.

MSC-0.3 exposure was accompanied by increased intracellular Ca^2+^ fluorescence, whereas no comparable increase was observed following NPS 2143 pretreatment. These exploratory cell-level findings were consistent with possible CaSR involvement but do not establish a causal mechanism. MSC-0.3 treatment was also associated with increased nuclear YAP localization, although the relationships among calcium signaling, YAP activation, and odontogenic responses remain to be established. Previous studies have reported that intracellular Ca^2+^ mobilization can reduce YAP phosphorylation and facilitate its nuclear translocation. Moreover, YAP activation has been associated with the regulation of genes involved in odontogenic differentiation [23,44].

ZrO_2_ was incorporated as a radiopacifying component and showed no detectable phase transformation during MSC hydration. However, radiopacity was not measured in the present study, and its radiographic performance remains to be established. The SP- and TEA-containing MSC-0.3 formulation, prepared at a nominal added-water-to-total-powder ratio of 0.30, exhibited greater early penetration resistance and compressive-strength development than the additive-free MSC-0.5 formulation. Because the amount of added water and the additive composition differed simultaneously between the formulations, the individual contributions of reduced water content, SP, and TEA could not be distinguished. The absolute strength values are specific to the present 5-mm cubic specimen geometry and should not be directly equated with values obtained using standardized cylindrical specimens; however, the internal comparison among formulations remains informative because all materials were tested under the same conditions. Standardized solubility, dimensional stability, radiopacity, and initial and final setting-time measurements are planned as part of dedicated application-specific validation; the 5-min Vicat measurement was used only for formulation screening.

Taken together, the present findings establish proof-of-concept feasibility for an M-S-H-forming dental cement platform rather than application-specific or clinical validation. Dedicated standards-based testing, broader biological evaluation, and in vivo studies are warranted before clinical translation.

## 5. Conclusions

Within the scope of this proof-of-concept study, the selected MSC-0.3 formulation formed an M-S-H-rich matrix, maintained moderated alkalinity, developed progressive mechanical strength, and supported favorable early hDPC responses. These findings support further investigation of MSC as an experimental dental biomaterial platform; however, standardized application-specific testing, antimicrobial evaluation, and in vivo validation remain necessary before clinical translation can be considered.

## Figures and Tables

**Table 1:** Chemical composition of reactive silica (RS).

Chemical Composition [wt%]	
Formula	RS
CaO	1.54
SiO_2_	96.90
Al_2_O_3_	0.29
MgO	0.18
Fe_2_O_3_	0.15
K_2_O	0.64
Na_2_O	0.16
P_2_O_5_	0.05

**Table 2:** Mixture proportions of the magnesium silicate-based cement (MSC).

Binder	ZrO_2_ [g]	MgO [g]	RS [g]	SP * [g]	TEA * [g]	Water [g]
MSC-0.5	30.0	14.0	56.0	-	-	50.0
MSC-0.3	30.0	14.0	56.0	5.5	0.5	30.0

* The formulation labels indicate the nominal added-water-to-total-powder ratio, calculated using the combined mass of MgO, RS, and ZrO_2_. SP and TEA were not included in this ratio and were dissolved in the separately added deionized water before mixing.

## Data Availability

The data that support the findings of this study are available from the corresponding author upon reasonable request.

## Supplementary Materials

The following supporting information can be downloaded at: https://www.mdpi.com/article/10.3390/jfb17100502/s1, Table S1. Descriptive statistics for quantitative physicochemical and biological outcomes. Table S2. Omnibus ANOVA results for quantitative outcomes. Figure S1. Representative SEM–EDS analyses of MSC-0.3 and ProRoot MTA after 3 and 14 days of hydration.

## Abbreviations

The following abbreviations are used in this manuscript:

MSC	Magnesium silicate-based cement
MTA	Mineral trioxide aggregate
M-S-H	Magnesium silicate hydrate
RS	Reactive silica
SP	Superplasticizer
TEA	Triethanolamine
XRD	X-ray diffraction
DTG	Differential thermogravimetry
SEM	Scanning electron microscopy
EDS	Energy-dispersive X-ray spectroscopy
hDPCs	Human dental pulp cells
ALP	Alkaline phosphatase
MTT	3-(4,5-Dimethylthiazol-2-yl)-2,5-diphenyltetrazolium bromide
YAP	Yes-associated protein
CaSR	Calcium-sensing receptor
GM	Growth medium
OM	Odontogenic differentiation medium
FBS	Fetal bovine serum
α-MEM	Alpha-minimum essential medium
