# Supplementary material for: Magnesium Silicate-Based Cement with Moderated Alkalinity: Physicochemical Characterization and Human Dental Pulp Cell Responses
Source: J Funct Biomater. 2026 Oct 4;17(10):502. doi: 10.3390/jfb17100502 (PMC13705994; doi:10.3390/jfb17100502)
Supplement: Supplementary file 1 [file jfb-17-00502-s001.zip › jfb-4538364-supplementary.pdf]

Table S1. Descriptive statistics for quantitative physicochemical and biological outcomes.

Outcome	Timepoint	Subgroup	Group	Unit	n	Mean ± SD
Compressive strength	Day 1		ProRoot MTA	MPa	3	15.52 ± 4.92
Compressive strength	Day 3		ProRoot MTA	MPa	3	60.29 ± 13.16
Compressive strength	Day 7		ProRoot MTA	MPa	3	61.33 ± 12.86
Compressive strength	Day 28		ProRoot MTA	MPa	3	65.33 ± 8.33
Compressive strength	Day 1		MSC-0.5	MPa	3	7.80 ± 0.32
Compressive strength	Day 3		MSC-0.5	MPa	3	7.62 ± 1.41
Compressive strength	Day 7		MSC-0.5	MPa	3	12.66 ± 2.78
Compressive strength	Day 28		MSC-0.5	MPa	3	14.29 ± 2.80
Compressive strength	Day 1		MSC-0.3	MPa	3	5.73 ± 0.84
Compressive strength	Day 3		MSC-0.3	MPa	3	14.14 ± 5.30
Compressive strength	Day 7		MSC-0.3	MPa	3	32.64 ± 3.90
Compressive strength	Day 28		MSC-0.3	MPa	3	41.88 ± 2.95
pH	Day 0		ProRoot MTA	pH units	3	10.97 ± 0.16
pH	Day 3		ProRoot MTA	pH units	3	12.04 ± 0.21
pH	Day 7		ProRoot MTA	pH units	3	12.08 ± 0.18
pH	Day 14		ProRoot MTA	pH units	3	12.11 ± 0.10
pH	Day 21		ProRoot MTA	pH units	3	12.10 ± 0.14
pH	Day 0		MSC-0.3	pH units	3	9.67 ± 0.16
pH	Day 3		MSC-0.3	pH units	3	9.61 ± 0.13
pH	Day 7		MSC-0.3	pH units	3	9.77 ± 0.18
pH	Day 14		MSC-0.3	pH units	3	8.98 ± 0.17
pH	Day 21		MSC-0.3	pH units	3	8.90 ± 0.15
5-min Vicat penetration	5 min		MSC-0.3	mm	3	0.30 ± 0.00
5-min Vicat penetration	5 min		MSC-0.5	mm	3	2.27 ± 0.40
5-min Vicat penetration	5 min		ProRoot MTA	mm	3	1.07 ± 0.06
Cell viability	24 h	1:1	Control	% of untreated control	3	100.00 ± 5.00
Cell viability	24 h	1:1	MSC-0.3	% of untreated control	3	86.00 ± 6.00
Cell viability	24 h	1:1	ProRoot MTA	% of untreated control	3	100.00 ± 6.00
Cell viability	24 h	1:2	Control	% of untreated control	3	100.00 ± 5.00
Cell viability	24 h	1:2	MSC-0.3	% of untreated control	3	90.00 ± 5.00
Cell viability	24 h	1:2	ProRoot MTA	% of untreated control	3	90.00 ± 5.00
Cell viability	24 h	1:4	Control	% of untreated control	3	100.00 ± 5.00
Cell viability	24 h	1:4	MSC-0.3	% of untreated control	3	112.00 ± 7.00
Cell viability	24 h	1:4	ProRoot MTA	% of untreated control	3	119.00 ± 4.00
Cell viability	24 h	1:8	Control	% of untreated control	3	100.00 ± 5.00

Outcome	Timepoint	Subgroup	Group	Unit	n	Mean ± SD
Cell viability	24 h	1:8	MSC-0.3	% of untreated control	3	97.00 ± 6.00
Cell viability	24 h	1:8	ProRoot MTA	% of untreated control	3	95.00 ± 4.00
Cell viability	72 h	1:1	Control	% of untreated control	3	100.00 ± 5.00
Cell viability	72 h	1:1	MSC-0.3	% of untreated control	3	63.00 ± 4.00
Cell viability	72 h	1:1	ProRoot MTA	% of untreated control	3	100.00 ± 6.00
Cell viability	72 h	1:2	Control	% of untreated control	3	100.00 ± 5.00
Cell viability	72 h	1:2	MSC-0.3	% of untreated control	3	80.00 ± 5.00
Cell viability	72 h	1:2	ProRoot MTA	% of untreated control	3	86.00 ± 5.00
Cell viability	72 h	1:4	Control	% of untreated control	3	100.00 ± 5.00
Cell viability	72 h	1:4	MSC-0.3	% of untreated control	3	93.00 ± 5.00
Cell viability	72 h	1:4	ProRoot MTA	% of untreated control	3	90.00 ± 5.00
Cell viability	72 h	1:8	Control	% of untreated control	3	100.00 ± 5.00
Cell viability	72 h	1:8	MSC-0.3	% of untreated control	3	103.00 ± 4.00
Cell viability	72 h	1:8	ProRoot MTA	% of untreated control	3	80.00 ± 5.00
Cell viability	7 d	1:1	Control	% of untreated control	3	100.00 ± 5.00
Cell viability	7 d	1:1	MSC-0.3	% of untreated control	3	45.00 ± 5.00
Cell viability	7 d	1:1	ProRoot MTA	% of untreated control	3	76.00 ± 8.00
Cell viability	7 d	1:2	Control	% of untreated control	3	100.00 ± 5.00
Cell viability	7 d	1:2	MSC-0.3	% of untreated control	3	53.00 ± 4.00
Cell viability	7 d	1:2	ProRoot MTA	% of untreated control	3	80.00 ± 4.00
Cell viability	7 d	1:4	Control	% of untreated control	3	100.00 ± 5.00
Cell viability	7 d	1:4	MSC-0.3	% of untreated control	3	104.00 ± 6.00
Cell viability	7 d	1:4	ProRoot MTA	% of untreated control	3	90.00 ± 4.00
Cell viability	7 d	1:8	Control	% of untreated control	3	100.00 ± 5.00
Cell viability	7 d	1:8	MSC-0.3	% of untreated control	3	89.00 ± 6.00
Cell viability	7 d	1:8	ProRoot MTA	% of untreated control	3	64.00 ± 6.00
ALP activity	Day 3		Control	Relative activity / total protein	3	1.000 ± 0.150
ALP activity	Day 3		MSC-0.3	Relative activity / total protein	3	2.000 ± 0.170
ALP activity	Day 3		ProRoot MTA	Relative activity / total protein	3	1.830 ± 0.131
ALP activity	Day 7		Control	Relative activity / total protein	3	1.000 ± 0.150
ALP activity	Day 7		MSC-0.3	Relative activity / total protein	3	2.550 ± 0.180
ALP activity	Day 7		ProRoot MTA	Relative activity / total protein	3	1.950 ± 0.120
ALP activity	Day 14		Control	Relative activity / total protein	3	1.000 ± 0.150
ALP activity	Day 14		MSC-0.3	Relative activity / total protein	3	3.000 ± 0.190
ALP activity	Day 14		ProRoot MTA	Relative activity / total protein	3	2.750 ± 0.140
YAP localization	Cytoplasmic		GM control	Normalized YAP level	3	1.000 ± 0.000
YAP localization	Cytoplasmic		OM	Normalized YAP level	3	0.887 ± 0.035
YAP localization	Cytoplasmic		MSC-0.3	Normalized YAP level	3	0.510 ± 0.040

Outcome	Timepoint	Subgroup	Group	Unit	n	Mean ± SD
YAP localization	Cytoplasmic		ProRoot MTA	Normalized YAP level	3	0.823 ± 0.035
YAP localization	Nuclear		GM control	Normalized YAP level	3	1.000 ± 0.000
YAP localization	Nuclear		OM	Normalized YAP level	3	1.370 ± 0.040
YAP localization	Nuclear		MSC-0.3	Normalized YAP level	3	2.263 ± 0.080
YAP localization	Nuclear		ProRoot MTA	Normalized YAP level	3	1.503 ± 0.055

Note. Vicat penetration is presented descriptively because it was used as a formulation-screening endpoint. Values are calculated from independent batch or donor means, not from pooled technical replicates. YAP values were normalized to the corresponding GM control within each donor; therefore, the GM-control donor mean was 1.000 for all donors.

Table S2. Omnibus ANOVA results for quantitative outcomes.

Note. Partial η^2 was calculated as $(F \times df_1)/(F \times df_1 + df_2)$. Cell-viability factorial analyses included MSC-0.3 and ProRoot MTA only; untreated controls were excluded from the material × dilution omnibus analyses.

Outcome	Analysis	Effect	df	F	Exact p	Partial η²
Compressive strength	Two-way ANOVA	Material	2, 24	119.503	3.334×10^-13	0.909
Compressive strength	Two-way ANOVA	Hydration time	3, 24	39.331	2.012×10^-9	0.831
Compressive strength	Two-way ANOVA	Material × hydration time	6, 24	10.389	1.072×10^-5	0.722
pH	Two-way ANOVA	Material	1, 20	1825.251	3.962×10^-21	0.989
pH	Two-way ANOVA	Immersion time	4, 20	14.614	9.793×10^-6	0.745
pH	Two-way ANOVA	Material × immersion time	4, 20	35.256	8.526×10^-9	0.876
Cell viability (24 h)	Two-way repeated-measures ANOVA	Material	1, 2	43.383	0.0223	0.956
Cell viability (24 h)	Two-way repeated-measures ANOVA	Dilution	3, 6	1738.508	3.318×10^-9	0.999
Cell viability (24 h)	Two-way repeated-measures ANOVA	Material × dilution	3, 6	70.647	4.517×10^-5	0.972
Cell viability (72 h)	Two-way repeated-measures ANOVA	Dilution	3, 6	1391.854	6.459×10^-9	0.999
Cell viability (72 h)	Two-way repeated-measures ANOVA	Material × dilution	3, 6	2045.088	2.039×10^-9	0.999
Cell viability (72 h)	Two-way repeated-measures ANOVA	Material	1, 2	96.213	0.0102	0.980
Cell viability (7 d)	Two-way repeated-measures ANOVA	Material	1, 2	1094.286	9.126×10^-4	0.998
Cell viability (7 d)	Two-way repeated-measures ANOVA	Dilution	3, 6	625.769	7.065×10^-8	0.997
Cell viability (7 d)	Two-way repeated-measures ANOVA	Material × dilution	3, 6	568.873	9.394×10^-8	0.996
ALP activity	Two-way repeated-measures ANOVA	Material	2, 4	3021.043	4.377×10^-7	0.999
ALP activity	Two-way repeated-measures ANOVA	Day	2, 4	9651.782	4.292×10^-8	>0.999
ALP activity	Two-way repeated-measures ANOVA	Material × day	4, 8	5528.242	8.550×10^-14	>0.999
Nuclear YAP	One-way repeated-measures ANOVA	Condition	3, 6	499.020	1.389×10^-7	0.996
Cytoplasmic YAP	One-way repeated-measures ANOVA	Condition	3, 6	108.223	1.298×10^-5	0.982

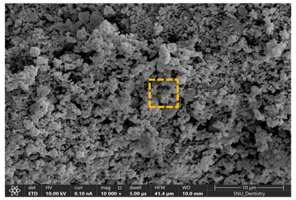

MSC-0.3, day 3

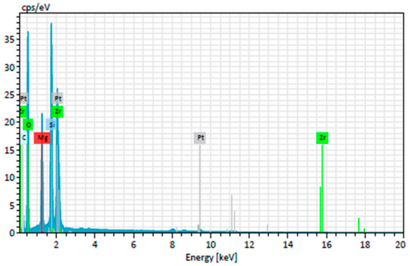

Element	Mass [%]	Mass Norm. [%]	Atom [%]
C	12.20	11.60	19.26
O	51.94	49.40	61.54
Mg	8.93	8.49	6.96
Si	11.94	11.35	8.06
Zr	20.13	19.15	4.18
Pt	0.00	0.00	0.00
	105.13	100.00	100.00

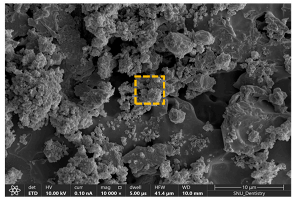

ProRoot MTA, day 3

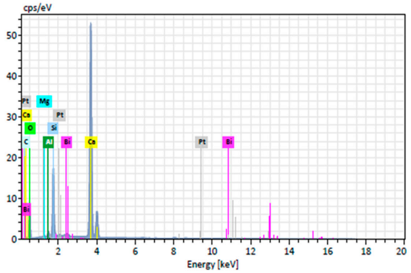

Element	Mass [%]	Mass Norm. [%]	Atom [%]
C	1.29	3.69	7.16
O	12.97	37.18	54.11
Al	0.50	1.44	1.25
Si	6.61	18.94	15.70
Ca	12.89	36.96	21.47
Mg	0.05	0.14	0.13
Pt	0.00	0.00	0.00
Bi	0.57	1.63	0.18
	34.88	100.00	100.00

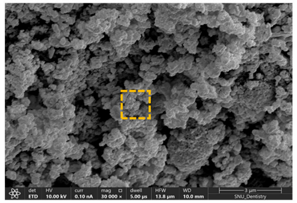

MSC-0.3, day 14

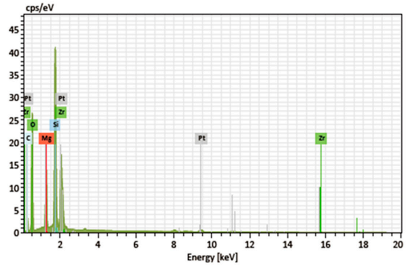

Element	Mass [%]	Mass Norm. [%]	Atom [%]
C	11.53	13.24	21.44
O	40.89	46.94	57.09
Mg	7.72	8.86	7.09
Si	14.10	16.19	11.22
Zr	12.87	14.78	3.15
Pt	0.00	0.00	0.00
	87.11	100.00	100.00

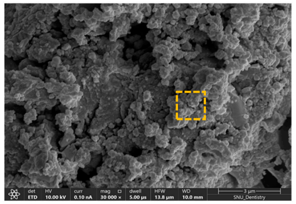

ProRoot MTA, day 14

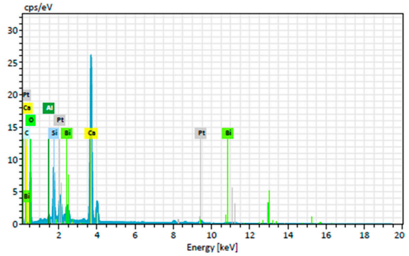

Element	Mass [%]	Mass Norm. [%]	Atom [%]
C	3.54	9.25	17.36
O	15.09	39.43	55.59
Al	0.35	0.92	0.77
Si	3.97	10.36	8.32
Ca	11.48	29.99	16.88
Pt	0.00	0.00	0.00
Bi	3.84	10.04	1.08
	38.28	100.00	100.00

Figure S1. Representative SEM–EDS analyses of MSC-0.3 and ProRoot MTA after 3 and 14 days of hydration.

Yellow rectangles indicate the local regions used for EDS acquisition. MSC-0.3 exhibited Mg–Si–O-rich elemental profiles with detectable Zr, whereas ProRoot MTA showed Ca–Si–O-rich profiles with detectable Bi. Elemental compositions are presented as mass percentages, normalized mass percentages, and atomic percentages. The local Mg/Si atomic ratios of MSC-0.3 were approximately 0.86 and 0.63 at days 3 and 14, respectively. The spectra represent local measurements and do not establish bulk phase stoichiometry or definitive structural phase identity. Pt-related peaks were excluded from interpretation of the cement matrix composition.
